# Study on Mechanism of Iridoid Glycosides Derivatives from *Fructus Gardeniae* in Jiangxi Province by Network Pharmacology

**DOI:** 10.1155/2020/4062813

**Published:** 2020-06-26

**Authors:** Fangzhou Liu, Yuanbai Li, Meng Li, Jing Wang, Yiying Zhang, Yu Du, Yang Yang

**Affiliations:** Institute of Information on Traditional Chinese Medicine, Beijing, China

## Abstract

**Objective:**

To investigate the pharmacological mechanism of the iridoid glycosides from *Fructus Gardeniae* in Jiangxi province by network pharmacology. To provide a valuable research strategy for the rational use and in-depth research and development of *Fructus Gardeniae* from Jiangxi.

**Method:**

Previous research results of our group show that the contents of iridoid glycosides in *Fructus Gardeniae* from Jiangxi province have a significant difference compared with other regions (*P* < 0.05). Based on our previous experimental results, this study selected six characteristic high-content bioactive iridoid glycosides components of *Fructus Gardeniae* from Jiangxi province as candidate components. TCMSP database was used to obtain the process parameters of absorption, distribution, metabolism, and excretion (ADME) of candidate components. PubChem and SWISS online database were used to predict the related targets. Cytoscape software was used to the construct compound-target-disease (C-T-D) network of the *Fructus Gardeniae* iridoid glycosides ingredients. Furthermore, the GO biological process analysis and the pathway enrichment analysis were carried out using the CTD online analysis platform; then, an illustrated network that contains the main “chemicals-targets-pathway (C-T-P)” was constructed to analyze main biological pathways for obtaining the deep mechanism of *Fructus Gardeniae* in Jiangxi.

**Results:**

6 iridoid glycosides, namely geniposide, gardenoside, geniposidic acid, genipin 1-gentiobioside, gardoside, and shanzhiside, from *Fructus Gardeniae* in Jiangxi province were obtained as candidate components through previous work and network pharmacology screening. 36 corresponding targets were acted, such as BCL2, MAPT, F2, BCL2L1, PRKCD, PRKCB, HIF1A, and PRKCA. These targets could joint in pathways, such as signaling by GPCR, neuroactive ligand-receptor interaction, inflammatory mediator regulation of TRP channels, and ion channel transport. Interestingly, these pathways were highly associated with liver diseases, neurological diseases, hypertension, neoplasms, hyperalgesia, and inflammation. Remarkably, we boldly speculate that the *Fructus Gardeniae* from Jiangxi province can play a pharmacological role in hepatic encephalopathy through regulating multiple signaling pathways in an integrated manner.

**Conclusion:**

The method based on system pharmacology could help to find the key targets of characteristic high-content chemical constituents of herb from different producing areas, the signaling pathway and disease network of TCM, and provide useful information and data support for giving a further study on traditional Chinese medicine resources in different regions of China.

## 1. Introduction

*Gardenia jasminoides* Ellis (Rubiaceae) is an evergreen shrub. The dried ripe fruits of *Gardenia jasminoides* have been recorded as *Fructus Gardeniae* (“zhi zi” in Chinese) in the Chinese Pharmacopoeia (2000–2015 edition) [1], and the fruits are used in traditional Chinese medicine because of their diuretic, antiphlogistic, anti-inflammatory, hemostatic, antipyretic, cholagogue, and laxative effects [2–4]. *Fructus Gardeniae* has been reported to protect the liver and gallbladder cells from infringement, treat cardiovascular and cerebrovascular diseases, and promote ligament cell proliferation and collagen synthesis effectively [5, 6]. Researchers found that the chemical composition of *Fructus Gardeniae* includes iridoid glycosides [5, 7], crocins [8], phenylpropanoids [9], glycoprotein [10], and polysaccharides. Among those, iridoid glycosides are the major active constituents in *Fructus Gardeniae* [11–13].

**Figure 1:**
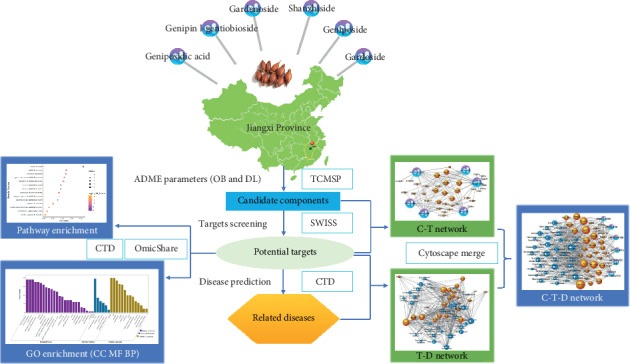
The schematic illustration of the network pharmacology analysis.

It is well known that traditional Chinese medicine (TCM) are normally collected from multiple geographical regions [14]. Accumulating studies have demonstrated that the place of origin could significantly affect the quality of TCM since climate and environment influence biosynthesis and accumulation of secondary metabolites in an organism [15]. The geographical origins of *Fructus Gardeniae* are also multiple. The contents of chemical constituents in *Fructus Gardeniae* from different habitats are different, and the different chemical constituents can regulate the subjects through multiple targets and pathways, thus producing different pharmacological effects. In our previous studies, *Fructus Gardeniae* cultivated in 10 different provinces in China has different high-content bioactive components and therefore, their different pharmacological activities. Preexperimental results demonstrated that the contents of geniposide, gardenoside, geniposidic acid, genipin 1-gentiobioside, gardoside, and shanzhiside in *Fructus Gardeniae* from Jiangxi province were significantly higher than those in other regions (*P* < 0.05) [16]; therefore, 6 iridoid glycosides were selected as the candidate components for the further pharmacological mechanism prediction. In this study, the key targets and signaling pathways of the characteristic high-content bioactive components group of *Fructus Gardeniae* from Jiangxi province were searched by network pharmacology, and the related diseases were predicted, and the chemicals-target-disease network was constructed. This study provides a basis for the in-depth study of the pharmacological mechanism of *Fructus Gardeniae* from Jiangxi province, China. The schematic illustration of this study is shown in Figure 1.

## 2. Materials and Methods

### 2.1. Candidate Components and ADME Parameters

Traditional Chinese Medicines for Systems Pharmacology Database and Analysis Platform (http://lsp.nwu.edu.cn/index.php, TCMSP) is a database of systems pharmacology for drug discovery from herbal medicines [17]. A growing number of systems pharmacology-based studies have recently been conducted in efforts to decipher the active mechanisms of TCM. Chen et al. predicted the potential mechanism of Yinchensini decoction based on systemic pharmacology by using TCMSP database. We followed the methods of Chen et al. [18]. From the TCMSP database, geniposide, gardenoside, geniposidic acid, genipin 1-gentiobioside, gardoside, and shanzhiside in *Fructus Gardeniae* from Jiangxi province are retrieved. And the process parameters of absorption, distribution, metabolism, and excretion (ADME) of candidate components were obtained by the TCMSP database. Each candidate's drug ability was analyzed according to its oral bioavailability (OB) and drug-likeness (DL) indices recommended by TCMSP. OB refers to the speed and degree of absorbing drugs into the circulatory system, which is a reliable indicator to evaluate the intrinsic quality of drugs objectively. The higher the OB of the compound is, the more likely the compound has the potential of being developed into drugs for clinical application [19]. DL refers to the comparative measure of the functional or physical properties of compounds with those of the majority of known drugs [20], which is the sum of the pharmacokinetic properties and safety. It can be used to analyze the results of the drug activity, predict in vivo pharmacokinetics, and optimize compounds. Bioactive molecules with high DL were often considered to exhibit relatively better pharmacologically.

### 2.2. Targets Screening

The targets prediction for the characteristic high-content bioactive components group of *Fructus Gardeniae* from Jiangxi province were performed using Swiss Target Prediction (http://www.swisstargetprediction.ch/) [21] by inputting the canonical SMILES into SMILES string (s) [22, 23], with the organism selected as “Homo sapiens”. The compound targets having no relationship with the compounds were deleted. Swiss is a free public resource to explore interactions between chemicals and gene targets. The potential targets are ranked by probability from high to low. And gene information including gene ID, name, and organism was identified using the UniProt database (http://www.uniprot.org/).

### 2.3. Chemicals-Target-Disease Network Construction

In this study, a “chemicals-target-disease (C-T-D)” network related to *Fructus Gardeniae* from Jiangxi province was constructed. In order to elucidate the relationship among candidate compounds, potential targets and related diseases, a “compound-target (C-T)” network, and a “disease-target (D-T)” network were generated for studying. (1) C-T network: compound-target interactions were visualized by the C-T network, in which all the active ingredients were connected to their corresponding targets. (2) T-D network: the target name was inputted to the CTD (http://ctdbase.org/) to predict the disease associated with the genes, diseases with genome frequency ≥1% were chosen and others were eliminated. Then, put the data into excel, plotting the network graph of the C-T network and D-T network with Cytoscape 3.7.0 software [24]. (3) C-T-D network: The merge function of Cytoscape software is used to generate the C-T network and the T-D network to construct a C-T-D network, that we used nodes to represent the compounds, targets, and diseases, and the lines between two nodes represented the interaction. We also used the degree to determine the size of each node and the betweenness to determine the thickness of lines and nodes and ensured that the graph would be obvious [25].

### 2.4. Gene Ontology and Pathway Analysis

The Comparative Toxicogenomics Database (http://ctdbase.org/, CTD) was adopted to conduct the Gene Ontology (GO) function analysis and the signaling pathway enrichment analysis. During this procedure, the significance level was set to 0.01, and organism was selected as Homo sapiens. The GO defines concepts related to gene function and the interrelationships among the functions of different genes [26]. It describes the functions of the candidate components in *Fructus Gardeniae* from Jiangxi province in terms of the molecular function, the cellular component involved, and the biological process affected [27]. In this study, pathway enrichment was additionally performed, for studying the biological effects and multidimensional pharmacological mechanism of *Fructus Gardeniae* from Jiangxi province at the pathway level. Then, an illustrated network that contains main “chemicals-targets-pathway (C-T-P)” of *Fructus Gardeniae* from Jiangxi province was constructed. The illustrated network that contains the main chemicals-targets-signaling pathway (C-T-P) of *Fructus Gardeniae* from Jiangxi province was established to understand their interaction. The results of the GO function analysis and the signaling pathway enrichment analysis were visualized via OmicShare platform2 (OmicShare, 2018).

## 3. Results

### 3.1. Target Identification of Candidate Components

The ADME parameters of candidate components obtained from TCMSP are shown in Table 1. Potential targets were predicted by SWISS servers. And based on the aforementioned target fishing approach, a total of 36 targets were predicted to interact with the 6 compounds identified in *Fructus Gardeniae* from Jiangxi province. The results of target name, gene name, UniProt ID, and matching probability are also shown in Table 1.

### 3.2. C-T-D Network Construction and Analysis

**Figure 2:**
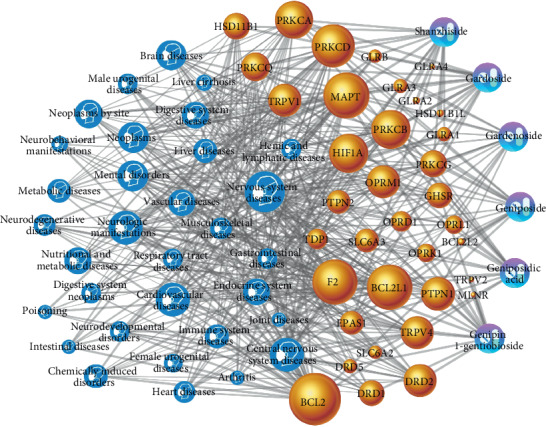
The C-T-D network generated in this study. Yellow nodes represent the potential targets, the purple capsule-shaped nodes represent the candidate compounds, and the blue head-shaped nodes represent the related diseases, while the edges represent the interactions between them.

As shown in Figure 2, a chemicals-target-disease (C-T-D) network model graph was constructed with 74 nodes (6 candidate components, 36 potential targets, and 32 related diseases) and 416 edges. In the network, the yellow nodes (36) represent the potential targets related to the candidate compounds; the purple capsule-shaped nodes (6) represent the candidate compounds in *Fructus Gardeniae* from Jiangxi province; and the blue head-shaped nodes (32) represent the related diseases to the targets, while the edges represent the interactions between them. The node size varies according to degree, and edge thickness varies according to betweenness. Results of the network topology analysis are as follows: network density (0.168), network heterogeneity (0.527), and shortest paths (5402, 100%). The average degree of nodes is 12.27027, and there are 34 nodes larger than the average degree. The average betweenness centrality of nodes is 0.01763, and there are 26 nodes larger than the average betweenness centrality. The key core nodes (target or disease) are screened based on the topological properties of the degree of network nodes. The results showed that the eight targets of BCL2, MAPT, F2, BCL2L1, PRKCD, PRKCB, HIF1A, and PRKCA were in the front of degree ranking (degree > 20), and they were the pivotal nodes in the network, indicating that they might be the core targets of the pharmacological mechanism of candidate components; among the disease nodes, the nervous system diseases, cardiovascular diseases, mental disorders, neoplasms, brain diseases, endocrine system diseases, nutritional and metabolic diseases, digestive system diseases, chemically induced disorders, immune system diseases, metabolic diseases, and liver diseases rank ahead, suggesting that the candidate components may play a pharmacological role in these diseases.

### 3.3. Analysis of GO Enrichment

**Figure 3:**
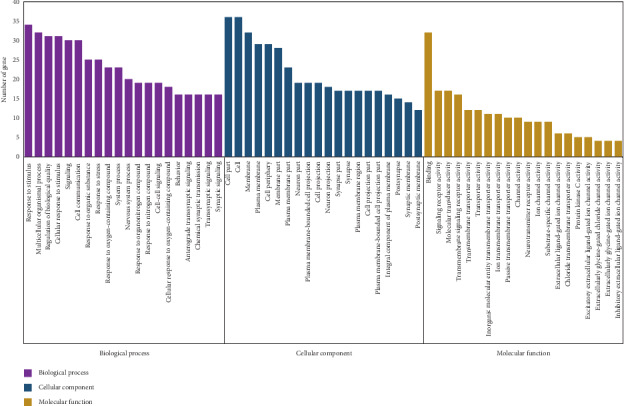
GO enrichment of 36 target genes. The horizontal axis represents the categories of “biological process,” “cellular components,” and “molecular functions” in the GO of the target genes, while the vertical axis represents the significant enrichment counts of these terms.

As highlighted in Figure 3, the gene ontology enrichment analysis consisted of three parts, BP (biological process), CC (cellular component), and MF (molecular function). Different categories of biological process, cellular component, and molecular function were represented by a purple, blue, and yellow bar, respectively. The height of the bar represented the number of genes observed in the category.

The enrichment results showed that there were 445 enrichment processes related to the biological processes, which cover response to the oxygen-containing compound, regulation of biological quality, response to organonitrogen compound, response to nitrogen compound, chemical synaptic transmission, and anterograde transsynaptic signaling; 73 enrichment results in the related items of cell composition, involving neuron projection, synapse part, neuron part, plasma membrane region, and postsynapse; 58 enrichment results are related to molecular function, which includes extracellularly the glycine-gated ion channel activity, the extracellularly glycine-gated chloride channel activity, the neurotransmitter receptor activity, protein phosphatase 2A binding, and chloride transmembrane transporter activity. Each *P* value of enrichment results was calculated (*P* values < 0.01 were considered to be significantly enriched), ranking *P* values according to the order from small to large. The top 20 enrichment results are displayed, and details are shown in Tables 2–4.

### 3.4. Analysis of Pathway

**Figure 4:**
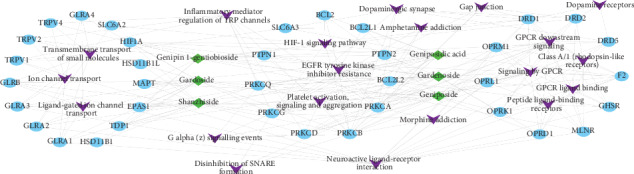
The main chemicals-targets-signaling pathway (C-T-P) of *Fructus Gardeniae* from Jiangxi province. Target genes are shown with blue circular nodes, green diamond nodes represent the characteristic high-content bioactive components of *Fructus Gardeniae* from Jiangxi province, and purple *V* nodes are signaling pathways.

**Figure 5:**
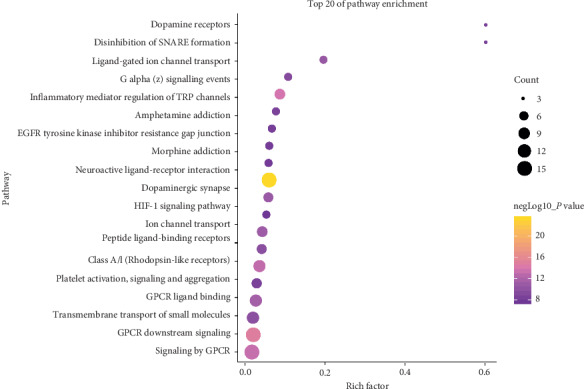
Pathway enrichment point diagram of 36 target genes. The vertical axis represents the pathway name, the horizontal axis represents the rich factor, the size of the dot indicates the number of genes expressed in the pathway, and the color of the dot corresponds to the different *P* value range.

The pathway analysis result showed that 33 of the 36 (91.6%) potential targets were enriched and involved 181 signaling pathways, and 84 of these pathways were significantly correlated with the target genes (*P* < 0.01). The top 20 pathway with lower *P* values and more genes enrichment are listed in Table 5, including signaling by GPCR, neuroactive ligand-receptor interaction, inflammatory mediator regulation of TRP channels, ion channel transport, dopaminergic synapse, amphetamine addiction, disinhibition of SNARE formation, EGFR tyrosine kinase inhibitor resistance, morphine addiction, and the HIF-1 signaling pathway. For the 20 listed pathways, 8 of them belonged to signal transduction, 3 belonged to human diseases, 3 belonged to transport of small molecules, 2 belonged to organismal systems, 2 belonged to hemostasis, 1 belonged to signaling molecules and interaction, while, the last 1 belonged to cellular processes. These pathways mainly involve liver diseases (liver cirrhosis and chemical- and drug-induced liver injury), neurological diseases (amphetamine-related disorders, cocaine-related disorders, autistic disorder, and schizophrenia), hypertension, neoplasms, hyperalgesia, inflammation, cardiomegaly, asthma, and diabetes mellitus. The illustrated network that contains the main chemicals-targets-signaling pathway (C-T-P) of *Fructus Gardeniae* from Jiangxi province was established to understand their interaction (Figure 4). The senior bubble map visually showed these significantly enriched pathways (Figure 5). The size and color of the nodes in the bubble graph were decided by the number of associated genes and the *P* values. The size of the nodes indicated how many target genes are associated, and the colors from purple to yellow reflected the *P* values from high to low.

## 4. Discussion

Bioactive components in TCM are the material basis to ensure its quality. From the biological point of view, the formation of the original plant varieties of TCM can be seen as the product of the interaction between genotype and habitat environment. The efficacy of TCM is closely related to its geographical origin. Newly Revised Materia Medica (Xin xiu ben cao), a materia medica commissioned by the government of the Tang Dynasty, stated: “if medicinal material is not produced from its native environment, the effect will be different.” That is to say, different geographical regions are suitable for growing different medicinal materials, and different medicinal materials also have different adaptability to the ecological environment of different producing areas. China has a vast territory, spanning tropical, subtropical, temperate, subfrigid, and plateau climate zones, and its terrain is complex. The conditions of sunshine, temperature, precipitation, and soil vary greatly in different regions, thus forming a complex and diverse ecosystem. There are also differences in the properties, chemical composition, and pharmacodynamics of traditional Chinese medicines bred in different ecosystems. The research on the correlation between the origin of TCM and the content of its chemical constituents has always been a worldwide scientific research hotspot in the field of traditional Chinese medicine resources. However, there are few experimental studies on pharmacodynamics of TCM from different geographical origins. The reason is that the chemical composition system of TCM is very complex. The contents of chemical constituents in medicinal materials from different habitats are different, and different chemical constituents can regulate the subjects through multiple targets and pathways, thus producing different pharmacological effects. Based on the above reasons, it is very difficult to carry out pharmacological experimental studies on traditional Chinese medicines from different origins because a large number of screening experiments need to be carried out from different geographical origins and different pharmacological effects. In the previous study, we collated the content data of *Fructus Gardeniae* from different geographical origins in scientific research literatures, and the content data were comprehensively evaluated by the single factor analysis of variance, principal component analysis, and cluster analysis to obtain the characteristic high-content bioactive components group in *Fructus Gardeniae* from different geographical origins. Based on network pharmacology, this study explored the key targets, signaling pathways, and disease networks of characteristic high-content chemical constituent groups of TCM from different geographical origins. The application of the network pharmacology method in the field of traditional Chinese medicine resources can solve the current research problems and provide powerful support for the research on pharmacological mechanism of traditional Chinese medicine from different geographical origins in China.

Preexperimental results demonstrated that among the 10 different provinces (Jiangxi, Sichuan, and Zhejiang, etc.) in China, the contents of six iridoid glycosides (geniposide, gardenoside, geniposidic acid, genipin 1-gentiobioside, gardoside, and shanzhiside) in *Fructus Gardeniae* from Jiangxi province were significantly higher than those in other regions (*P* < 0.05). Then, these six iridoid glycosides constitute the characteristic high-content chemical constituent group of *Fructus Gardeniae* from Jiangxi province. Iridoid glycosides have been demonstrated to be the major bioactive ingredients in *Fructus Gardeniae* [28–30], among which, geniposide is the quality control standard recorded in the Chinese Pharmacopoeia (2000–2015 edition) [31], and gardenoside as a hydroxylation product of geniposide is also an important constituent in *Fructus Gardeniae* [5, 6]. This is similar to those recorded in ancient Chinese materia medica books. Jiangxi province is traditionally considered to be the “daodi” production region for *Fructus Gardeniae* (zhi zi), that is, *Fructus Gardeniae* from Jiangxi province is considered as “daodi medicinal materials” and is called “Jiang zhi zi” in Chinese. The term “daodi medicinal material” refers to a concept that has been widely recognized in the field of TCM for centuries. It is defined as “medicinal material that is produced and assembled in specific geographical regions with designated natural conditions and ecological environment [32]. These factors lead to quality and clinical effects surpass those of same botanical origin produced from other geographical regions, and thus, it is widely recognized and has long enjoyed a good reputation [33].” Although it is also known as genuine medicinal material, geo-authentic medicinal material, authentic and superior medicinal herbal, authentic medicinal, and geoherb [34–36]. Based on our previous experimental results, this study selected the six characteristic high-content bioactive components of *Fructus Gardeniae* from Jiangxi province as candidate components, using Swiss Target Prediction to obtain 36 potential targets related to candidate components, and imported 18 targets into CTD database to predict target-related diseases. 32 diseases with a genome frequency ≥1% were selected to construct the C-T-D network. The nodes in the network ranked according to degree. The results showed that the eight targets of BCL2, MAPT, F2, BCL2L1, PRKCD, PRKCB, HIF1A, and PRKCA were in the front of degree ranking (degree >20), and among the disease nodes, the nervous system diseases, cardiovascular diseases, mental disorders, neoplasms, brain diseases, endocrine system diseases, nutritional and metabolic diseases, digestive system diseases, chemically induced disorders, immune system diseases, metabolic diseases, and liver diseases rank ahead, suggesting that characteristic high-content bioactive components group (geniposide, gardenoside, geniposidic acid, genipin 1-gentiobioside, gardoside, and shanzhiside) of *Fructus Gardeniae* from Jiangxi province may play a pharmacological role in these diseases through the potential targets such as BCL2, MAPT, F2, BCL2L1, and PRKCD. That is to say, in the field of traditional Chinese medicine resources, if we want to carry out pharmacodynamic experimental research on *Fructus Gardeniae* from Jiangxi province, we should start with antinervous diseases, anticardiovascular diseases, antimental disorders, antineoplasms, antibrain diseases, and antiendocrine system diseases; if we want to carry out the related pharmacological mechanism experimental research, we can start with BCL2, MAPT, F2, BCL2L1, PRKCD, PRKCB, HIF1A, PRKCA, and other 25 potential targets.

In this study, the GO enrichment analysis and the pathway enrichment analysis were carried out in order to explore the multidimensional pharmacological mechanism of the characteristic high-content bioactive components group of *Fructus Gardeniae* from Zhejiang province. The results showed that the pathways with higher number of related annotated genes count and lower the *P* value mainly included signaling by GPCR, neuroactive ligand-receptor interaction, inflammatory mediator regulation of TRP channels, ion channel transport, dopaminergic synapse, amphetamine addiction, disinhibition of SNARE formation, EGFR tyrosine kinase inhibitor resistance, morphine addiction, and HIF-1 signaling pathway. Among these pathways, the GPCR pathway, platelet activation signaling-and-aggregation, and ion channel transport are mainly associated with liver cirrhosis and hypertension; neuroactive ligand-receptor interaction, dopamine receptors, dopaminergic synapse, amphetamine addiction, and morphine addiction are mainly associated with neurological diseases such as autism, schizophrenia, and cocaine-related diseases; EGFR tyrosine kinase inhibitor resistance, gap junction, and the HIF-1 signaling pathway are mainly associated with neoplasms and liver cirrhosis; inflammatory mediator regulation of TRP channels is mainly associated with hyperalgesia, inflammation, and liver cirrhosis; ligand-gated ion channel transport is mainly associated with autistic disorder and brain injuries. From the above analysis, we find an interesting phenomenon that among these diseases related to high-enrichment pathways, neurological diseases (including amphetamine-related disorders, cocaine-related disorders, autistic disorder, and schizophrenia.) and liver diseases (including liver cirrhosis, carcinoma hepatocellular, and chemical- and drug-induced liver injury.) seem to present a particularly high frequency. These results make us naturally associate with a complex disease, which is “hepatic encephalopathy”. Hepatic encephalopathy (HE) is a known neurologic complication of advanced cirrhosis [37]. Between 30% and 50% of hospitalization cases of cirrhosis are related to HE [38]. HE is a syndrome of spectrum of neuropsychiatric abnormalities caused by portosystemic venous shunting, with intrinsic liver disease. Related neurocomorbidities include depression, bipolar disorder, schizophrenia, drug abuse, and suicidal tendencies [39]. On the basis of the results obtained in this study, we boldly speculate that the *Fructus Gardeniae* from Jiangxi province can play a pharmacological role in hepatic encephalopathy through regulating multiple signaling pathways in an integrated manner.

This study reveals the related targets, key biological pathways, and main disease types of the characteristic high-content chemical constituent group of *Fructus Gardeniae* from Jiangxi province from the perspective of network pharmacology. In conclusion, our study applied the network pharmacology method to the field of traditional Chinese medicine resources innovatively, and it laid a foundation for the study of multidimensional pharmacological mechanism of *Fructus Gardeniae* from different geographical regions in China.

## Figures and Tables

**Table 1:** Information of potential targets from characteristic high-content bioactive components group of *Fructus Gardeniae* from Jiangxi province in China.

Molecule	OB (%)	DL	Target	Gene	UniProt ID	Probability^*∗*^(%)
Geniposide	14.64	0.44	MX2 : AA81X2 : AA81microtubule-associated protein tau	MAPT	P10636	75.00
	14.64	0.44	Protein kinase C gamma type	PRKCG	P05129	24.00
	14.64	0.44	Protein kinase C beta type	PRKCB	P05771	24.00
	14.64	0.44	Protein kinase C alpha type	PRKCA	P17252	24.00
	14.64	0.44	Protein kinase C theta type	PRKCQ	Q04759	24.00
	14.64	0.44	Protein kinase C delta-type regulatory subunit	PRKCD	Q05655	24.00
	14.64	0.44	Hypoxia-inducible factor 1-alpha	HIF1A	Q16665	24.00
	14.64	0.44	Endothelial PAS domain-containing protein 1	EPAS1	Q99814	24.00
	14.64	0.44	Bcl-2-like protein 1	BCL2L1	Q07817	18.00
	14.64	0.44	Apoptosis regulator Bcl-2	BCL2	P10415	18.00
	14.64	0.44	Bcl-2-like protein 2	BCL2L2	Q92843	18.00
	14.64	0.44	Mu-type opioid receptor	OPRM1	P35372	14.00
	14.64	0.44	Delta-type opioid receptor	OPRD1	P41143	14.00
	14.64	0.44	Kappa-type opioid receptor	OPRK1	P41145	14.00
	14.64	0.44	Nociceptin receptor	OPRL1	P41146	14.00

Gardenoside	52.77	0.12	Microtubule-associated protein tau	MAPT	P10636	68.00
	52.77	0.12	Protein kinase C gamma type	PRKCG	P05129	25.00
	52.77	0.12	Protein kinase C beta type	PRKCB	P05771	25.00
	52.77	0.12	Protein kinase C alpha type	PRKCA	P17252	25.00
	52.77	0.12	Protein kinase C theta type	PRKCQ	Q04759	25.00
	52.77	0.12	Protein kinase C delta-type regulatory subunit	PRKCD	Q05655	25.00
	52.77	0.12	Hypoxia-inducible factor 1-alpha	HIF1A	Q16665	16.00
	52.77	0.12	Endothelial PAS domain-containing protein 1	EPAS1	Q99814	16.00
	52.77	0.12	Tyrosine-protein phosphatase nonreceptor type 2	PTPN2	P17706	16.00
	52.77	0.12	Tyrosine-protein phosphatase nonreceptor type 1	PTPN1	P18031	16.00
	52.77	0.12	Tyrosyl-DNA phosphodiesterase 1	TDP1	Q9NUW8	15.00
	52.77	0.12	Mu-type opioid receptor	OPRM1	P35372	14.00
	52.77	0.12	Delta-type opioid receptor	OPRD1	P41143	14.00
	52.77	0.12	Kappa-type opioid receptor	OPRK1	P41145	14.00
	52.77	0.12	Nociceptin receptor	OPRL1	P41146	14.00

Geniposidic acid	19.59	0..41	Microtubule-associated protein tau	MAPT	P10636	74.00
	19.59	0..41	Hypoxia-inducible factor 1-alpha	HIF1A	Q16665	29.00
	19.59	0..41	Endothelial PAS domain-containing protein 1	EPAS1	Q99814	29.00
	19.59	0..41	Protein kinase C gamma type	PRKCG	P05129	22.00
	19.59	0..41	Protein kinase C beta type	PRKCB	P05771	22.00
	19.59	0..41	Protein kinase C alpha type	PRKCA	P17252	22.00
	19.59	0..41	Protein kinase C theta type	PRKCQ	Q04759	22.00
	19.59	0..41	Protein kinase C delta-type regulatory subunit	PRKCD	Q05655	22.00
	19.59	0..41	Tyrosyl-DNA phosphodiesterase 1	TDP1	Q9NUW8	21.00
	19.59	0..41	Bcl-2-like protein 1	BCL2L1	Q07817	18.00
	19.59	0..41	Apoptosis regulator Bcl-2	BCL2	P10415	18.00
	19.59	0..41	Bcl-2-like protein 2	BCL2L2	Q92843	18.00
	19.59	0..41	Sodium-dependent noradrenaline transporter	SLC6A2	P23975	14.00
	19.59	0..41	Sodium-dependent dopamine transporter	SLC6A3	Q01959	14.00
	19.59	0..41	Activation peptide fragment 1	F2	P00734	14.00

Genipin 1-gentiobioside	45.58	0.83	Microtubule-associated protein tau	MAPT	P10636	71.00
	45.58	0.83	Hypoxia-inducible factor 1-alpha	HIF1A	Q16665	31.00
	45.58	0.83	Endothelial PAS domain-containing protein 1	EPAS1	Q99814	31.00
	45.58	0.83	Tyrosine-protein phosphatase nonreceptor type 2	PTPN2	P17706	20.00
	45.58	0.83	Tyrosine-protein phosphatase nonreceptor type 1	PTPN1	P18031	20.00
	45.58	0.83	Transient receptor potential cation channel subfamily V member 1	TRPV1	Q8NER1	14.00
	45.58	0.83	Activation peptide fragment 1	F2	P00734	13.00
	45.58	0.83	Transient receptor potential cation channel subfamily V member 4	TRPV4	Q9HBA0	13.00
	45.58	0.83	Transient receptor potential cation channel subfamily V member 2	TRPV2	Q9Y5S1	13.00
	45.58	0.83	Tyrosyl-DNA phosphodiesterase 1	TDP1	Q9NUW8	11.00
	45.58	0.83	D (1A) dopamine receptor	DRD1	P21728	10.00
	45.58	0.83	D (1B) dopamine receptor	DRD5	P21918	10.00
	45.58	0.83	Motilin receptor	MLNR	O43193	10.00
	45.58	0.83	Growth hormone secretagogue receptor type 1	GHSR	Q92847	10.00
	45.58	0.83	D (2) dopamine receptor	DRD2	P14416	9.00

Gardoside	—	—	Microtubule-associated protein tau	MAPT	P10636	86.00
	—	—	Hypoxia-inducible factor 1-alpha	HIF1A	Q16665	22.00
	—	—	Endothelial PAS domain-containing protein 1	EPAS1	Q99814	22.00
	—	—	Glycine receptor subunit alpha-1	GLRA1	P23415	19.00
	—	—	Glycine receptor subunit alpha-2	GLRA2	P23416	19.00
	—	—	Glycine receptor subunit alpha-3	GLRA3	O75311	19.00
	—	—	Glycine receptor subunit beta	GLRB	P48167	19.00
	—	—	Glycine receptor subunit alpha-4	GLRA4	Q5JXX5	19.00
	—	—	Corticosteroid 11-beta-dehydrogenase isozyme 1	HSD11B1	P28845	12.00
	—	—	Hydroxysteroid 11-beta-dehydrogenase 1-like protein	HSD11B1L	Q7Z5J1	12.00
	—	—	Tyrosyl-DNA phosphodiesterase 1	TDP1	Q9NUW8	12.00
	—	—	Protein kinase C gamma type	PRKCG	P05129	10.00
	—	—	Protein kinase C beta type	PRKCB	P05771	10.00
	—	—	Protein kinase C alpha type	PRKCA	P17252	10.00
	—	—	Protein kinase C theta type	PRKCQ	Q04759	10.00

Shanzhiside	117.77	0.10	Microtubule-associated protein tau	MAPT	P10636	89.00
	117.77	0.10	Hypoxia-inducible factor 1-alpha	HIF1A	Q16665	19.00
	117.77	0.10	Endothelial PAS domain-containing protein 1	EPAS1	Q99814	19.00
	117.77	0.10	Tyrosyl-DNA phosphodiesterase 1	TDP1	Q9NUW8	14.00
	117.77	0.10	Protein kinase C alpha type	PRKCA	P17252	14.00
	117.77	0.10	Protein kinase C delta-type regulatory subunit	PRKCD	Q05655	14.00
	117.77	0.10	Protein kinase C gamma type	PRKCG	P05129	14.00
	117.77	0.10	Protein kinase C beta type	PRKCB	P05771	14.00
	117.77	0.10	Protein kinase C theta type	PRKCQ	Q04759	14.00
	117.77	0.10	Corticosteroid 11-beta-dehydrogenase isozyme 1	HSD11B1	P28845	14.00
	117.77	0.10	Hydroxysteroid 11-beta-dehydrogenase 1-like protein	HSD11B1L	Q7Z5J1	14.00
	117.77	0.10	Glycine receptor subunit alpha-1	GLRA1	P23415	10.00
	117.77	0.10	Glycine receptor subunit alpha-2	GLRA2	P23416	10.00
	117.77	0.10	Glycine receptor subunit alpha-3	GLRA3	O75311	10.00
	117.77	0.10	Glycine receptor subunit beta	GLRB	P48167	10.00

**Table 2:** Gene ontology term, biological process, direct (top 20).

GO term	*P* value	Count
Regulation of biological quality	1.19*E* − 28	31
Response to oxygen-containing compound	6.99*E* − 25	23
Response to organonitrogen compound	1.16*E* − 22	19
Response to nitrogen compound	4.74*E* − 22	19
System process	1.03*E* − 21	23
Response to stimulus	1.44*E* − 21	34
Nervous system process	4.39*E* − 21	20
Behavior	5.52*E* − 21	16
Anterograde transsynaptic signaling	7.06*E* − 21	16
Chemical synaptic transmission	7.06*E* − 21	16
Transsynaptic signaling	8.08*E* − 21	16
Multicellular organismal process	8.74*E* − 21	32
Response to organic substance	8.89*E* − 21	25
Synaptic signaling	9.48*E* − 21	16
Signaling	1.59*E* − 20	30
Cell communication	1.75*E* − 20	30
Cellular response to stimulus	3.39*E* − 20	31
Cellular response to oxygen-containing compound	6.00*E* − 20	18
Cell-cell signaling	9.99*E* − 19	19
Response to stress	1.04*E* − 18	25

**Table 3:** Gene ontology term, cellular component, direct (top 20).

GO term	*P* value	Count
Plasma membrane	5.61*E* − 21	29
Cell periphery	9.05*E* − 21	29
Plasma membrane part	1.3*E* − 20	23
Synapse part	5.5*E* − 20	17
Synaptic membrane	5.9*E* − 20	14
Neuron projection	1.12*E* − 19	18
Postsynapse	1.68*E* − 19	15
Membrane	6.59*E* − 19	32
Neuron part	9.05*E* − 19	19
Synapse	2.89*E* − 18	17
Plasma membrane region	5.72*E* − 18	17
Postsynaptic membrane	8.24*E* − 18	12
Membrane part	3.92*E* − 17	28
Plasma membrane-bounded cell projection	4.77*E* − 17	19
Cell projection part	8.26*E* − 17	17
Plasma membrane-bounded cell projection part	8.26*E* − 17	17
Cell projection	8.95*E* − 17	19
Integral component of plasma membrane	2.79*E* − 16	16
Cell part	4.92*E* − 16	36
Cell	5.74*E* − 16	36

**Table 4:** Gene ontology term, molecular function, direct (top 20).

GO term	*P* value	Count
Neurotransmitter receptor activity	2.98*E* − 17	9
Signaling receptor activity	1.05*E* − 16	17
Molecular transducer activity	1.64*E* − 16	17
Transmembrane signaling receptor activity	2.03*E* − 16	16
Channel activity	4.08*E* − 13	10
Extracellularly glycine-gated chloride channel activity	4.13*E* − 13	4
Extracellularly glycine-gated ion channel activity	4.13*E* − 13	4
Passive transmembrane transporter activity	4.19*E* − 13	10
Protein kinase C activity	1.33*E* − 12	5
Inhibitory extracellular ligand-gated ion channel activity	2.06*E* − 12	4
Transmembrane transporter activity	2.1*E* − 12	12
Inorganic molecular entity transmembrane transporter activity	2.1*E* − 12	11
Ion transmembrane transporter activity	5.42*E* − 12	11
Ion channel activity	6.29*E* − 12	9
Transporter activity	6.4*E* − 12	12
Binding	7.45*E* − 12	32
Substrate-specific channel activity	8.01*E* − 12	9
Extracellular ligand-gated ion channel activity	1.31*E* − 11	6
Excitatory extracellular ligand-gated ion channel activity	3.59*E* − 11	5
Chloride transmembrane transporter activity	4.64*E* − 11	6

**Table 5:** Classification and information on target components-related pathways, direct (top 20).

Classification	Pathway	*P* value	Annotated genes count
Signaling molecules and interaction	Neuroactive ligand-receptor interaction	4.25*E* − 24	15
Signal transduction	GPCR downstream signaling	1.17*E* − 15	15
	Signaling by GPCR	6.64*E* − 14	15
	Class A/1 (rhodopsin-like receptors)	9.8*E* − 14	10
	GPCR ligand-binding	2.95*E* − 12	10
	Peptide ligand-binding receptors	1.98*E* − 10	7
	G alpha (*z*) signaling events	5.01*E* − 10	5
	Dopamine receptors	5.3*E* − 09	3
	HIF-1 signaling pathway	2.24*E* − 08	5
Organismal systems	Inflammatory mediator regulation of TRP channels	1.38*E* − 14	8
	Dopaminergic synapse	1.47*E* − 11	7
Human diseases	Amphetamine addiction	3.01*E* − 09	5
	EGFR tyrosine kinase inhibitor resistance	6.47*E* − 09	5
	Morphine addiction	1.32*E* − 08	5
Cellular processes	Gap junction	1.12*E* − 08	5
Transport of small molecules	Ion channel transport	7.04*E* − 12	8
	Ligand-gated ion channel transport	1.95*E* − 11	5
	Transmembrane transport of small molecules	1.44*E* − 10	10
Hemostasis	Platelet activation, signaling, and aggregation	3.32*E* − 09	7
	Disinhibition of SNARE formation	5.3*E* − 09	3

## Data Availability

The data used to support the findings of our research are included within the article and the supplementary information files.

## Supplementary Materials

Supplementary Materials Supplementary 1. Table S1: the analysis data of chemicals-target (C-T) network. Supplementary 2. Table S2: the analysis data of target-disease (T-D) network. Supplementary 3. Table S3: the analysis data of targets-signaling pathway (T-P) network.
